# Application of non-Gaussian water diffusional kurtosis imaging in the assessment of uterine tumors: A preliminary study

**DOI:** 10.1371/journal.pone.0188434

**Published:** 2017-11-27

**Authors:** Aliou Amadou Dia, Masatoshi Hori, Hiromitsu Onishi, Makoto Sakane, Takashi Ota, Takahiro Tsuboyama, Mitsuaki Tatsumi, Tomoyuki Okuaki, Noriyuki Tomiyama

**Affiliations:** 1 Department of Diagnostic and Interventional Radiology, Osaka University Graduate School of Medicine, Suita, Japan; 2 Department of Radiology, Osaka University Hospital, Suita, Japan; 3 Philips Healthcare, Tokyo, Japan; Suzhou University, CHINA

## Abstract

**Objectives:**

To evaluate the interobserver reliability and value of diffusional kurtosis imaging (DKI) in the assessment of uterine tumors compared with those of conventional diffusion-weighted imaging (DWI).

**Methods:**

This retrospective study was approved by our institutional review board, which waived the requirement for informed consent. Fifty-eight women (mean age: 55.0 ± 13.6 years; range: 30–89 years) with suspected malignant uterine tumors underwent 3-T magnetic resonance imaging using DKI and DWI. Twelve had coexisting leiomyoma. Two observers analyzed region-of-interest measurements of diffusivity (D), kurtosis (K), and the apparent diffusion coefficient (ADC) of uterine lesions and healthy adjacent tissues. Interobserver agreement was evaluated using the intra-class correlation coefficient (ICC). The mean values were compared using one-way analysis of variance with a *post-hoc* Tukey’s honestly significant difference test. The diagnostic accuracy of D and ADC in differentiating malignant tumors from benign leiomyomas was analyzed using receiver operating characteristic (ROC) analysis.

**Results:**

The ICCs between the two observers in evaluating D, K, and the ADC of the malignant tumors were higher than 0.84, suggesting excellent interobserver agreements. The mean D (×10^−3^ mm^2^/s) of uterine cancers (1.05 ± 0.41 and 1.09 ± 0.40 for observers 1 and 2, respectively) were significantly lower than those of leiomyoma (1.40 ± 0.37 and 1.56 ± 0.33, respectively; *P* < 0.05), healthy myometrium (1.72 ± 0.27 and 1.69 ± 0.30, respectively; *P* < 0.001), and healthy endometrium (1.53 ± 0.35 and 1.42 ± 0.37, respectively; *P* < 0.005). There was no significant difference in the area under the ROC curve between D and ADC. The mean K of uterine cancers (0.88 ± 0.28 and 0.90 ± 0.23, respectively) were higher than those of myometrium (0.72 ± 0.10 and 0.73 ± 0.10, respectively; *P* < 0.001), healthy endometrium (0.65 ± 0.13 and 0.60 ± 0.18, respectively; *P* < 0.001), and leiomyoma (0.76 ± 0.14 and 0.77 ± 0.16, respectively; not significant, *P* > 0.1).

**Conclusions:**

Interobserver agreements in evaluating D, K, and ADC were moderate to excellent. D performed equally to conventional DWI in differentiating between benign and malignant uterine lesions. The mean K of malignant uterine lesions was significantly higher than that of non-tumorous myometrium or endometrium.

## Introduction

Uterine cancers include cervical cancers and corpus cancers. Cervical cancer is the second most common cancer in women worldwide with nearly 530,000 new cases and 275,000 deaths attributed to the disease annually [1]. Corpus cancers, also referred to as endometrial cancers, are the most commonly diagnosed gynecologic malignancy among women in the US; an estimated 60,050 new cases and 10,470 deaths are expected in 2016 [2].

Over the last decade, several studies on functional magnetic resonance (MR) imaging have demonstrated the utility of functional MR imaging sequences, such as diffusion-weighted imaging (DWI), in the diagnosis, and pre- and post-operative assessment of uterine cancers [3–10]. Tumors are frequently more cellular than the tissue from which they originate, thus appearing to have a relatively high-signal intensity on DWI [3]. Conventional DWI studies the motion of water molecules assumed to undergo Gaussian diffusion. However, water in biologic tissues is restricted by its interactions with other molecules and cell membranes; therefore, the assumption of Gaussian water diffusion may be inadequate to describe the actual diffusion in tissues [11].

Diffusional kurtosis imaging (DKI) is a new and promising diffusion imaging technique that extends DWI through the quantification of non-Gaussian water diffusion and requires the use of multiple and higher *b* values. Recently, DKI was developed with the aim of characterizing the diffusional heterogeneity emerging from multiple tissue compartments with different diffusivities [11, 12]. DKI provides novel *in vivo* diffusion properties that describe tissue microstructure by analyzing not only diffusivity (D) but also kurtosis (K), which is a unit-less index of non-Gaussianity [13, 14]. DKI has been widely applied in recent years because of its clinical utility and robust theoretical framework [13, 15]. To the best of our knowledge, however, the utility of DKI for the evaluation of uterine tumors has not yet been investigated. The purpose of this study was to evaluate the interobserver reliability and the value of DKI in the assessment of uterine tumors compared with that of conventional DWI.

## Materials and methods

### Ethics statements

This retrospective study was approved by the Osaka University Hospital institutional review board (approval number: 14395), which waived the necessity for informed consent.

### Patients

We retrospectively searched the radiology database of our institution for patients with suspected malignant uterine tumors who underwent MR imaging, including multi-*b* value DWI, before therapy between November 15th, 2013 and February 4th, 2015. Fifty-eight women (mean age 55.0 ± 13.6 years [standard deviation], range 30–89 years) with suspected malignant uterine tumors were included in the study. After surgery (N = 26) or biopsy (N = 32), 53 had histopathologic proof of malignancy and the remaining five had no histopathologic proof of malignancy. For five of the 53 women who had histopathologically proven malignant tumors, it was difficult to measure signal intensity of the tumor on diffusion-weighted images because the tumor was too small to be observed on MR images. Of the 48 malignant tumors for which signal intensities could be measured on MR images, the most frequent lesions were squamous cell carcinoma of the uterine cervix (N = 24), adenocarcinoma of the uterine cervix (N = 9), and endometrioid adenocarcinoma of the uterine corpus (N = 5; Table 1). Twelve of the 58 women had typical leiomyomas, for which signal intensities could be measured on diffusion-weighted images.

**Table 1:** Histopathologic findings of uterine malignant tumors that can be measured on diffusion-weighted images.

Histopathologic findings of uterine malignant tumors	Number
Squamous cell carcinoma of the uterine cervix	24
Adenocarcinoma of the uterine cervix	9
Poorly differentiated carcinoma of the uterine cervix	3
Small cell carcinoma of the uterine cervix	1
Endometrioid adenocarcinoma of the uterine corpus	5
Mixed carcinoma of the uterine corpus (endometrioid adenocarcinoma and serous adenocaricnoma)	2
Clear cell adenocarcinoma of the uterine corpus	1
Adenosquamous carcinoma of the uterine corpus	1
Carcinosarcoma of the uterine corpus	2
Total	48 *

Note

*Of the 58 women, 5 had no histopathologic proof of malignancy and 53 had histopathologic proof of malignancy. For 5 of the 53 women who had histopathologically proven malignant tumors, it was difficult to measure signal intensity of the tumor on diffusion-weighted images because the tumor was too small to be observed on magnetic resonance images.

Twelve of the 58 women had coexisting leiomyoma.

### Magnetic resonance imaging protocol

A 3T MR scanner was used (Achieva 3.0T X; Philips Healthcare, Best, The Netherlands) using 32-channel torso/cardiac coils. Unless contraindicated, patients received 20 mg of intramuscular butylscopolamine to prevent artifacts resulting from peristalsis. A parallel imaging technique (SENSE) was used. Axial images of the pelvis were initially obtained using two-dimensional (2D) T2-weighted single-shot turbo spin-echo and three-dimensional T1-weighted gradient-echo MR imaging, with and without fat-suppression sequences. The patients then underwent T2-weighted imaging with 2D turbo spin-echo (TSE) sequences. TSE images were obtained at a thickness of 4 mm in both the parasagittal (parallel to the longitudinal axis of the uterus) and axial oblique (orthogonal to the longitudinal axis of the uterus) planes. DWI images were acquired using a single-shot spin-echo echo-planar sequence (*b* = 0, 700, 1000, 1700, and 2500 s/mm^2^) in the parasagittal planes (Table 2). We used a maximum *b* value of 2500 s/mm^2^ according to Jensen et al [11]. Dynamic contrast-enhanced images were obtained for all patients, but those images were not used in our study.

**Table 2:** Diffusional kurtosis imaging parameters.

*b* values (s/mm^2^)	Imaging plane	Acqusition Time (s)	Repetition Time / Echo Time (ms)	Flip Angle (deg)	Field of View (cm)	EPI factor	Fat saturated	Section Thickness / Gap (mm)	Matrix	No. of Slices	No. of Signals Acquired	Parallel Imaging Factor	Bandwidth (pixels)
**0,** **700,** **1000,** **1700,** **2500**	Parasagittal	420	4000 / 77	90	28	51	Yes	4 / 0	96 x 96	16	3	2	14

Note: Diffusional kurtosis imaging was acquired using a single-shot spin-echo echo-planar sequence.

### Image analysis

Using Philips Research Integrated Development Environment (PRIDE) software (Philips Healthcare), we conducted region-of-interest (ROI) measurements of D, K, and the apparent diffusion coefficient (ADC) of malignant tumors of the endometrium and uterine cervix, leiomyoma, healthy myometrium, and healthy endometrium. Two radiologists with different years of experience in abdominal imaging (9 and 5 years) independently drew circular ROIs for each lesion and adjacent non-tumorous regions (healthy myometrium and endometrium) at a *b* value of 0 s/mm^2^ in the region of highest contrast that was devoid of intratumoral necrosis. These ROIs were then automatically copied for other *b* values (*b* = 700, 1000, 1700, and 2500 s/mm^2^). The ROI areas ranged from 12.5 mm^2^ to 314.2 mm^2^. D and K were calculated using the following formula:

$$S=S_{0}∙e^{-b∙D+\frac{-b^{2}+D^{2}∙K}{6}}$$

where S and S_0_ represent the signal intensities of the images acquired at *b* and *b*_*0*_, respectively (s/mm^2^). D and K represent diffusivity (mm^2^ /s) and kurtosis, respectively. Standard ADC (mm^2^/s) was obtained using a conventional mono-exponential fit with the following equation:

$$S=S_{0}∙e^{-b∙ADC}$$

where S and S_0_ represent the signal intensities acquired at *b* and *b*_*0*_, respectively. DKI parameters (D and K) were calculated using *b* values ranging from 0 to 2500 s/mm^2^. The ADC was calculated using *b* values of 0 and 700 s/mm^2^.

### Statistical analysis

The intra-class correlation coefficient (ICC) (2,1) was used to assess interobserver agreement between the two radiologists. The mean values for uterine malignant tumor, leiomyoma, healthy myometrium, and healthy endometrium were calculated; the differences in mean values were tested using one-way analysis of variance (ANOVA) with a *post-hoc* Tukey’s honestly significant difference test. An unpaired t-test was used to evaluate the differences in D, K, and ADC between squamous cell carcinoma and adenocarcinoma. The diagnostic accuracy of D and the ADC in differentiating malignant tumors from benign leiomyomas was compared using DeLong’s test for two correlated receiver-operating characteristic (ROC) curves.

For all statistical analyses other than the ROC analysis, IBM SPSS software (Version 21: IBM, Somers, NY, USA) was used. For ROC analysis, R software (Version 3.2.2: R Foundation for Statistical Computing, Vienna, Austria) was used. A two-tailed *P* value of <0.05 was considered to indicate a significant difference.

Post-hoc power analysis using the two-sided independent t-test was performed to evaluate the statistical power for detecting significant differences in K between malignant tumors and leiomyomas or between squamous cell carcinomas and adenocarcinomas with the PS software package (version 3.1.2; http://biostat.mc.vanderbilt.edu/wiki/Main/PowerSampleSize).

## Results

**Fig 1:**
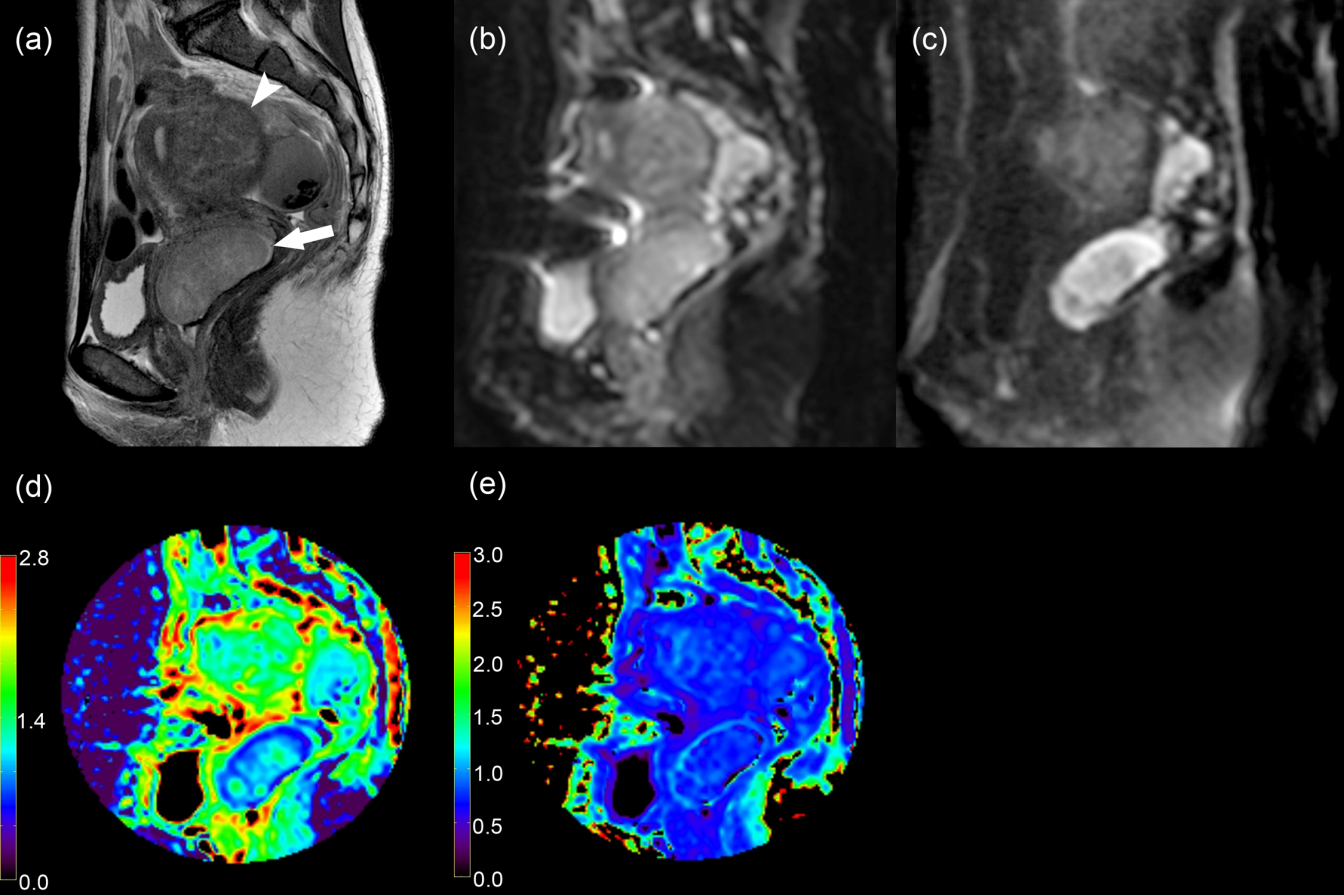
A 38-year-old woman with poorly differentiated carcinoma of the uterine cervix and coexisting posterior corporeal leiomyoma. (a) Sagittal T2-weighted image (TR/TE = 4500/80 ms), (b) sagittal diffusion-weighted image at *b* = 0 s/mm^2^, (c) sagittal diffusion-weighted image at *b* = 2500 s/mm^2^, (d) sagittal diffusivity map (D map), and (e) kurtosis map (K map) show the uterine cervical cancer (arrow). The tumor appears as a hyperintense mass on the diffusion-weighted image at *b* = 2500 s/mm^2^ with decreases in values on the D and K maps, whereas the leiomyoma (arrow head) has a low signal on the diffusion-weighted image, and an intermediate value on the D and K maps.

**Fig 2:**
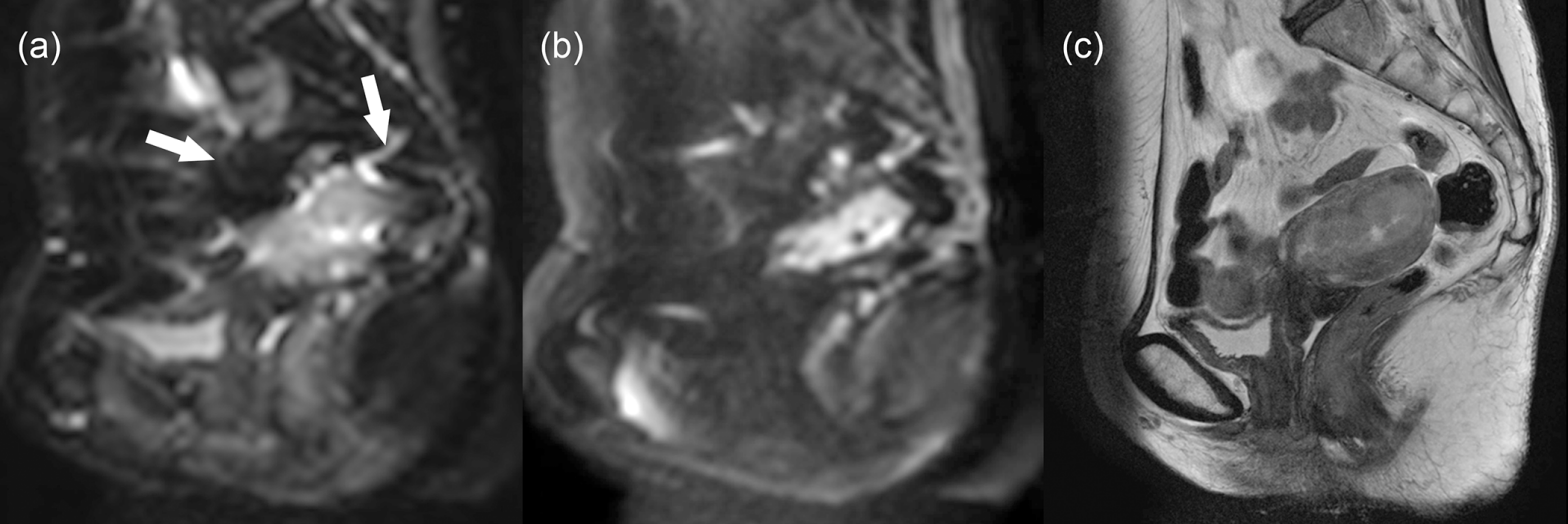
A 65-year-old woman with mixed carcinoma of the uterine corpus (serous adenocarcinoma, grade 2; endometrioid adenocarcinoma, grade 2). (a) Sagittal diffusion-weighted image at *b* = 0 s/mm^2^, (b) sagittal diffusion-weighted image at *b* = 2500 s/mm^2^, and (c) sagittal T2-weighted image show the tumor in the endometrium. Because of severe susceptibility artifacts due to bowel gas in the rectum and the sigmoid colon (arrows), it was hard to calculate diffusivity and kurtosis values for the myometrium. However, those values could be calculated for the endometrial tumor because of the lesser degree of artifacts at the center of the uterine corpus.

**Fig 3:**
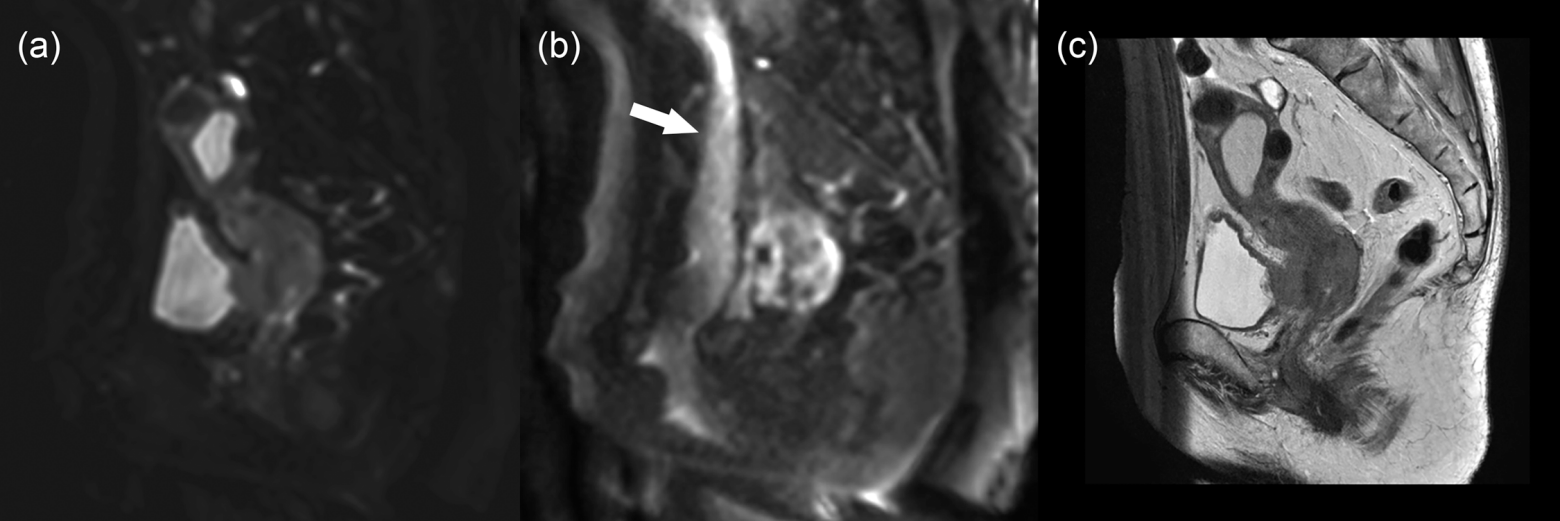
A 67-year-old woman with squamous cell carcinoma (keratinizing type) of the uterine cervix. (a) Sagittal diffusion-weighted image at *b* = 0 s/mm^2^, (b) sagittal diffusion-weighted image at *b* = 2500 s/mm^2^, and (c) sagittal T2-weighted image show the tumor in the uterine cervix. Because of a severe ghost artifact from the abdominal wall (arrow), it was hard to calculate diffusivity and kurtosis values for the myometrium. However, those values could be calculated for the cervical tumor because of the lesser degree of artifacts of the uterine cervix.

The DKI and ADC measurements were possible in 48 women with uterine malignant tumors, 12 with leiomyoma, 41 with healthy myometrium, and 19 with healthy endometrium. Fig 1 illustrates representative images from a woman with poorly differentiated carcinoma of the uterine cervix. Of the 58 women, D, K, and ADC values of uterine malignant tumors could not be measured in 10 because of (a) no pathological proof of malignancy (N = 5) or (b) no visible tumor on MR images (cervical cancer, 4; corpus cancer, 1). Values of healthy myometrium could not be measured in 17 women because of (a) small size of myometrium due to large tumors (N = 4), (b) small size of myometrium due to hydrometra or pyometra (N = 5), (c) high degree of susceptibility artifact associated with bowel gas (N = 7; Fig 2), and (d) severe abdominal wall ghosts (N = 1, Fig 3). Values of healthy endometrium could not be measured in 39 women because of (a) small size of healthy endometrium (N = 17), (b) small size of endometrium due to large tumors or endometrial hyperplasia (N = 9), (c) hydrometra or pyometra (N = 7), (d) high degree of susceptibility artifact associated with bowel gas (N = 4), and (e) severe abdominal wall ghosts (N = 2).

The ICCs between the two radiologists in evaluating D, K, and the ADCs of the 48 malignant tumors were higher than 0.84, suggesting excellent interobserver agreement (Table 3). The ICCs for leiomyoma, myometrium, and endometrium ranged from 0.43 to 0.87, suggesting moderate to excellent interobserver agreement (Table 3).

**Table 3:** Inter-rater reliability derived via the calculation of the intra-class correlation coefficients (ICC) of diffusivity (D), kurtosis (K), and the apparent diffusion coefficient (ADC) of uterine malignant tumor, leiomyoma, healthy myometrium, and healthy endometrium.

	D	K	ADC
Malignant tumor (N = 48)	0.92 (0.86, 0.95)	0.84 (0.73, 0.90)	0.86 (0.77, 0.92)
Leiomyoma (N = 12)	0.56 (0.06, 0.85)	0.72 (0.26, 0.91)	0.57 (0.07, 0.85)
Healthy myometrium (N = 41)	0.67 (0.47, 0.81)	0.74 (0.56, 0.85)	0.64 (0.41, 0.79)
Healthy endometrium (N = 19)	0.87 (0.58, 0.95)	0.43 (0.00, 0.73)	0.84 (0.64, 0.94)

Note: Data in parentheses are 95% confidence intervals.

**Fig 4:**
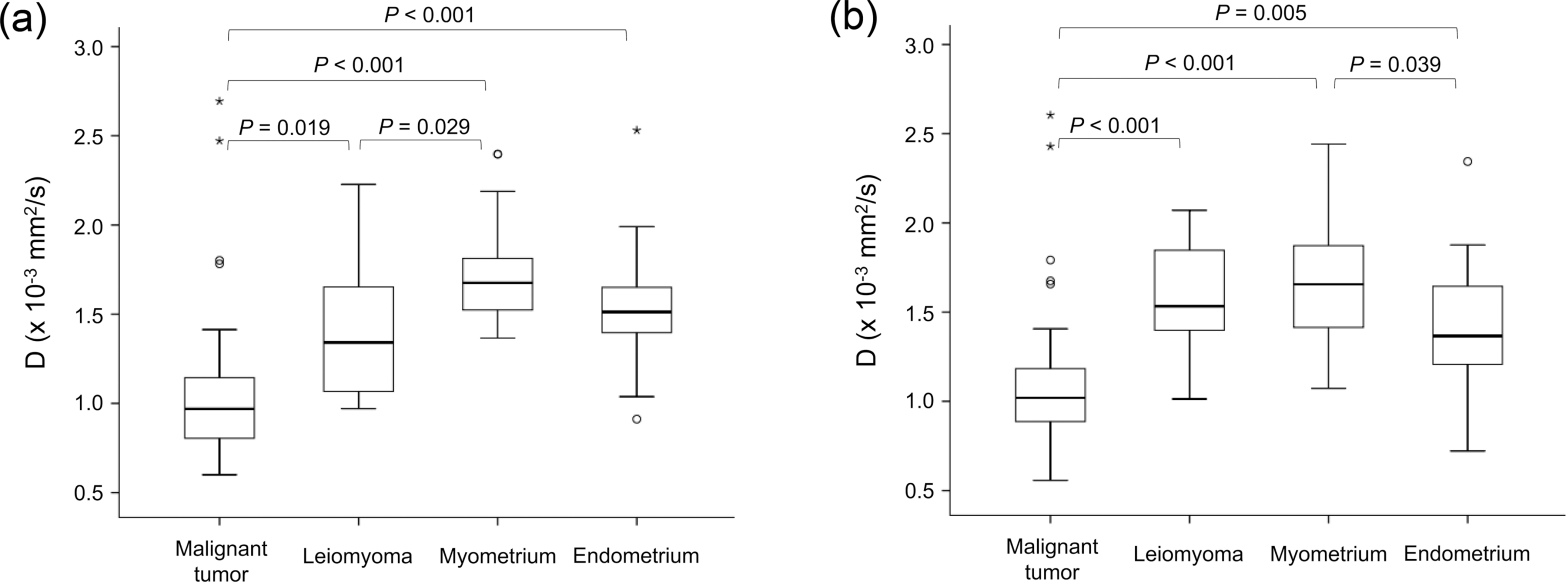
Box-and-whisker plot of mean diffusivity (D) of uterine malignant tumor, leiomyoma, healthy myometrium, and healthy endometrium for (a) observer 1 and (b) observer 2. Outliers are also represented (° and *). The top and bottom of each box represent the 25th and 75th percentiles of the mean D, respectively. The horizontal line inside each box represents the median value. The graphs show a significantly lower mean D for uterine malignant tumor than for leiomyoma, healthy myometrium, and healthy endometrium. There were no significant differences in D between leiomyoma and endometrium (*P* = 0.74), or myometrium and endometrium (*P* = 0.21) for observer 1. There were no significant differences in D between leiomyoma and myometrium (*P* = 0.71), or leiomyoma and endometrium (*P* = 0.70) for observer 2.

**Fig 5:**
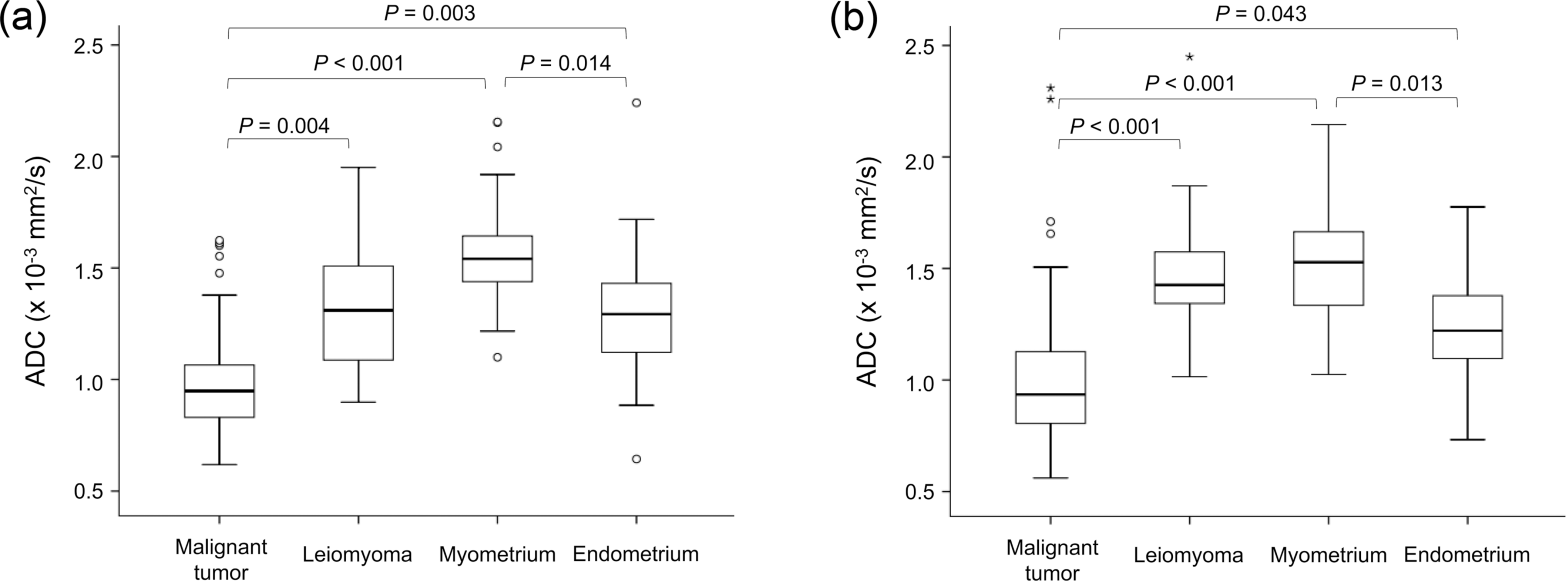
Box-and-whisker plot of the mean apparent diffusion coefficient (ADC) of uterine malignant tumor, leiomyoma, healthy myometrium, and healthy endometrium for (a) observer 1 and (b) observer 2. Outliers are also represented (° and *). The top and bottom of each box represent the 25th and 75th percentiles of the mean ADC, respectively. The horizontal line inside each box represents the median value. The graphs show a significantly lower mean ADC for uterine malignant tumor than for leiomyoma, healthy myometrium, and healthy endometrium. There were no significant differences in the ADC between leiomyoma and myometrium (*P* = 0.14 and 1.00 for observers 1 and 2, respectively), or leiomyoma and endometrium (*P* = 0.98 and 0.095, respectively).

**Fig 6:**
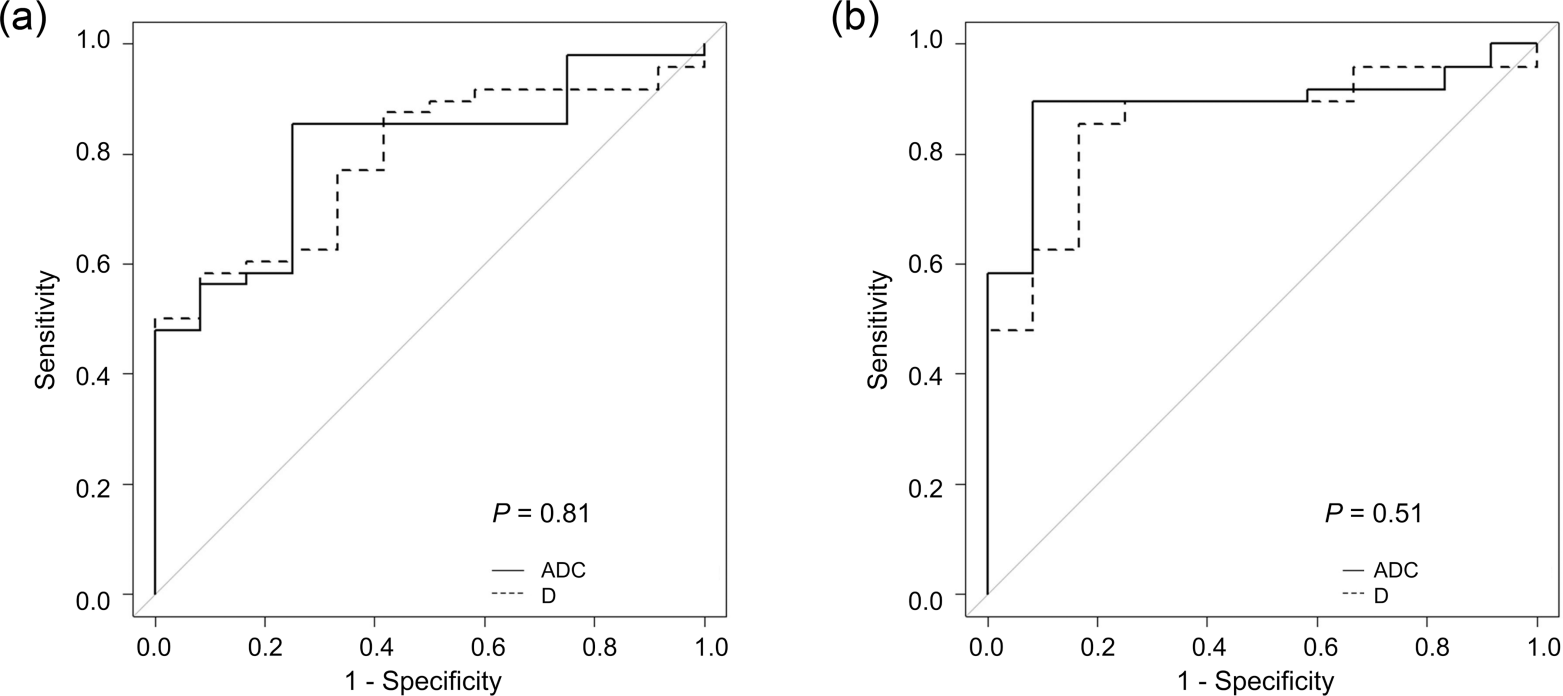
Receiver-operating characteristic curves for diffusivity (D, dashed line) and the apparent diffusion coefficient (ADC, solid line) to differentiate uterine malignant tumors from leiomyoma for (a) observer 1 and (b) observer 2. No significant differences in the area under the receiver-operating characteristic curve (AUC) were seen between D (0.79; 95% confidence interval (CI), 0.67–0.91) and the ADC (0.81; 95% CI, 0.68–0.93) for observer 1 (*P* = 0.81), or between D (0.86; 95% CI, 0.75–0.97) and ADC (0.89; 95% CI, 0.80–0.98) for observer 2 (*P* = 0.51).

The mean Ds (×10^−3^ mm^2^/s) of malignant tumors (1.05 ± 0.41 [standard deviation] and 1.09 ± 0.40 for observers 1 and 2, respectively) were significantly lower than those of leiomyoma (1.40 ± 0.37 and 1.56 ± 0.33, respectively), healthy myometrium (1.72 ± 0.27 and 1.69 ± 0.30, respectively), and healthy endometrium (1.53 ± 0.35 and 1.42 ± 0.37, respectively) for both observers (Fig 4). There were also significant differences between the mean Ds of leiomyoma and healthy myometrium for observer 1 (*P* = 0.029), and between the mean Ds of healthy myometrium and healthy endometrium for observer 2 (*P* = 0.039). In comparison, the mean ADCs (×10^−3^ mm^2^/s) of malignant tumors (1.00 ± 0.32 and 1.02 ± 0.36 for observers 1 and 2, respectively) were significantly lower than those of leiomyoma (1.34 ± 0.33 and 1.52 ± 0.36, respectively), healthy myometrium (1.55 ± 0.24 and 1.52 ± 0.25, respectively), and healthy endometrium (1.30 ± 0.34 and 1.25 ± 0.28, respectively) for both observers (Fig 5). There were also significant differences between the mean ADCs of healthy myometrium and healthy endometrium for both observers. A comparison of Figs 4 and 5 shows that the mean D and mean ADC had similar distributions for uterine lesions, healthy myometrium, and healthy endometrium. In ROC analysis, there was no significant difference in the area under the ROC curve between the mean D and mean ADC in terms of the accuracy of differentiation between malignant tumors and benign leiomyomas for both observers (Fig 6).

**Fig 7:**
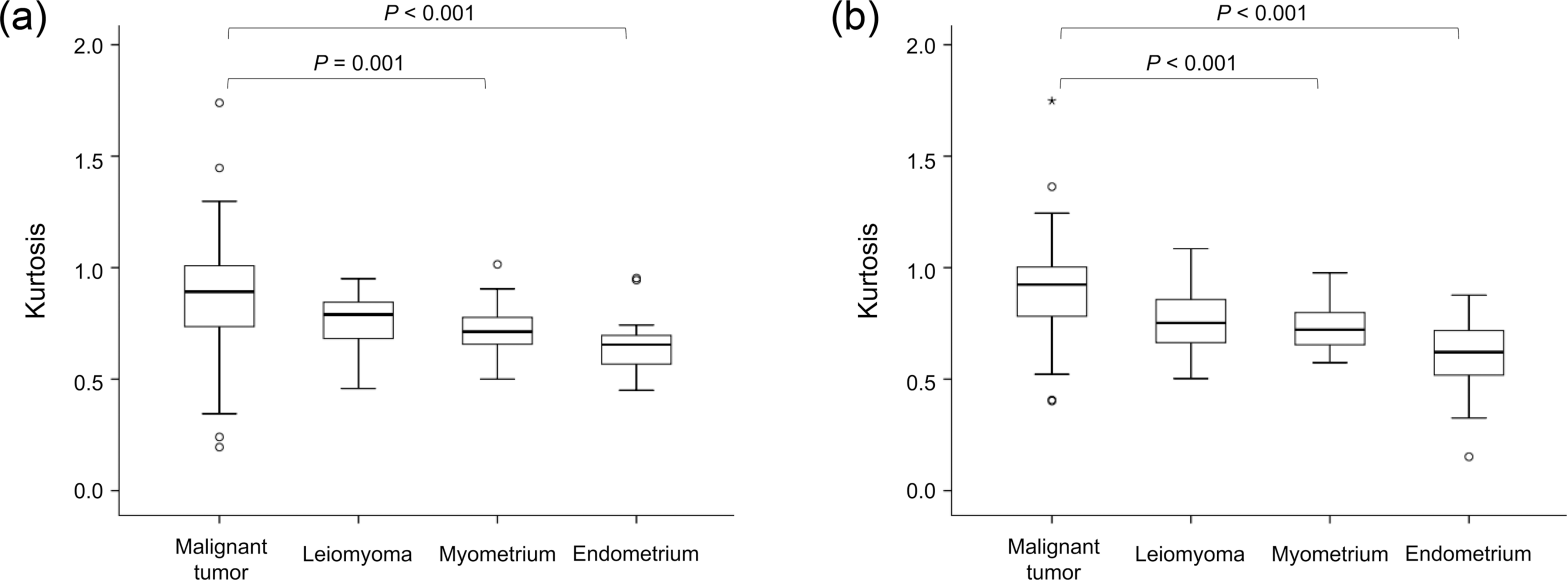
Box-and-whisker plot of mean kurtosis (K) of uterine malignant tumor, leiomyoma, healthy myometrium, and healthy endometrium for (a) observer 1 and (b) observer 2. Outliers are also represented (° and *). The top and bottom of each box represent the 25th and 75th percentiles of the mean K, respectively. The horizontal line inside each box represents the median value. The graphs show a significantly lower mean K for uterine malignant tumor than for healthy myometrium and healthy endometrium. There were no significant differences in K between malignant tumor and leiomyoma (*P* = 0.26 and 0.11 for observers 1 and 2, respectively); leiomyoma and myometrium (*P* = 0.92 and 0.93, respectively); leiomyoma and endometrium (*P* = 0.41 and 0.07, respectively); or myometrium and endometrium (*P* = 0.57 and 0.06, respectively).

The mean Ks of malignant tumors (0.88 ± 0.28 and 0.90 ± 0.23 for observers 1 and 2, respectively) were significantly higher than those of healthy myometrium (0.72 ± 0.10 and 0.73 ± 0.10, respectively) and healthy endometrium (0.65 ± 0.13 and 0.60 ± 0.18, respectively) for both observers (Fig 7). No significant differences were observed between the mean Ks of malignant tumors and those of leiomyoma (0.76 ± 0.14 and 0.77 ± 0.16, respectively), the mean Ks of leiomyoma and those of healthy myometrium and healthy endometrium, or the mean Ks of healthy myometrium and those of healthy endometrium, for either observer. The statistical powers for detecting significant differences in K between malignant tumors and leiomyomas were estimated as 0.25 and 0.40 for observers 1 and 2, respectively, suggesting that the sample size was not sufficient.

The Ds, ADCs, and Ks of squamous cell carcinoma of the uterine cervix (N = 24) were compared with those of adenocarcinoma (N = 17) (adenocarcinoma of the uterine cervix, 9; endometrioid adenocarcinoma of the uterine corpus, 5; mixed carcinoma of the uterine corpus, 2; and clear cell adenocarcinoma of the uterine corpus, 1). The mean D of squamous cell carcinomas (0.95 ± 0.18 and 0.98 ± 0.18 for observers 1 and 2, respectively) were significantly lower than those of adenocarcinoma (1.26 ± 0.59 and 1.32 ± 0.54, respectively) for both observers (*P* = 0.045 and 0.020, respectively). The mean ADCs of squamous cell carcinomas (0.89 ± 0.14 and 0.90 ± 0.16 for observers 1 and 2, respectively) were also significantly lower than those of adenocarcinoma (1.16 ± 0.42 and 1.25 ± 0.49, respectively) for both observers (*P* = 0.018 and 0.011, respectively). There were no significant differences between the mean Ks of squamous cell carcinoma (0.87 ± 0.22 and 0.89 ± 0.11, respectively) and adenocarcinoma (0.82 ± 0.29 and 0.82 ± 0.26, respectively) for either observer (*P* = 0.53 and 0.31, respectively). The statistical powers for detecting significant differences in K between squamous cell carcinomas and adenocarcinomas were estimated as 0.08 and 0.13 for observers 1 and 2, respectively.

## Discussion

Recently, several scientific studies have focused on DKI to attempt to elucidate the non-Gaussian properties of water diffusion in biologic and pathologic tissues, particularly in cerebrovascular stroke, demyelinating diseases, white matter aging, Parkinson’s disease, prostate cancer, breast cancer, and kidney cancer [14, 16–26]. However, the application of DKI for the uterus has not been well investigated. To our knowledge, there is only one recently published report that investigated the utility of various non-mono-exponential analyses of DWI including DKI for uterine cervical tumors [27]. In this study, we successfully performed DKI analysis of the uterus. We found that the mean Ds of cervical and endometrial cancers were significantly lower than those of leiomyoma, healthy myometrium, and healthy endometrium. ROC curve analyses (Fig 6) and a comparison among Figs 4 and 5 demonstrated equality in terms of diagnostic accuracy between D and the ADC.

Regarding the contribution of K, which relates to microstructural complexity, to the evaluation of uterine tumors, the mean K was higher in uterine cancers than in benign tissues such as the myometrium, endometrium, or leiomyoma. These findings suggest that diffusion in malignant uterine lesions shows greater deviation from Gaussian behavior compared with that in benign tissues, which is similar to that in breast and prostate cancers [19, 20, 28–30]. The differences were statistically significant between uterine cancer and either the myometrium or endometrium, although we were unable to demonstrate a statistically significant difference between cancer and leiomyoma. Because post-hoc power analysis suggested that the sample size was not sufficient, further studies with a larger sample size are definitely needed in terms of the role of K in differentiating between malignant and benign tissues. We also investigated the value of K in discriminating between different pathological types of uterine malignant tumors (i.e., squamous cell carcinoma and adenocarcinoma). There was no significant difference in mean K between the two types of malignant tumors. However, post-hoc power analysis suggested that the sample size was not sufficient; therefore, further studies with a larger sample size are needed for evaluating the role of K in differentiating between these types.

In terms of the DKI measurements, the interobserver agreements were good when the measurements were successful. Therefore, the application of DKI should be clinically feasible. However, it was sometimes impossible to measure the necessary values because of severe artifacts, as shown on Figs 2 and 3. As DWI with a high *b* value (such as 2500 s/mm^2^) has a tendency to have reduced signal intensity and stronger artifacts compared with those with lower *b*-value images, clinical application of the DKI technique may be limited due to the occurrence of stronger artifacts. Improving image quality for high *b*-value DWI would be crucial for the future use of DKI of the uterus. In addition to the problem of image quality, the longer acquisition time of DKI may be a problem in its routine use. Optimization of scan techniques to reduce acquisition time will also be important from the perspective of practical application [31].

Recently, Winfield et al. reported that DWI data analyses up to *b* value of 800 s/mm^2^ using non-mono-exponential models, such as stretched exponential, kurtosis, statistical, and bi-exponential models, were useful to distinguish between types and grades of uterine cervical tumors [27]. They noted that D was significantly different between tumor grades, and K was significantly different between squamous cell carcinomas and adenocarcinomas. However, as they described, the use of a maximum *b* value of 800 s/mm^2^ was a limitation of their study because the DKI model was originally developed over a much wider range of *b* values [11, 27]. Jensen et al. reported that the precision of DKI estimates rapidly decreased when the maximum *b* values were substantially reduced below 2000 s/mm^2^ in the brain [11]. In our study, we showed the feasibility of DKI analysis up to a higher *b* value of 2500 s/mm^2^ with interobserver reliability for uterine corpus and cervical tumors, leiomyomas, myometrium, and endometrium.

There are several limitations to this study. The first is related to its retrospective nature. The second is related to the limited number of participants, especially for evaluating the role of K. Third, the findings of this study are preliminary; further studies involving several centers and a larger sample size are necessary to better understand the value of DKI in the evaluation of uterine cancers. Fourth, in some cases, we were unable to perform the ROI measurement of the uterine tumor because of difficulties detecting or circumscribing the tumor. Fifth, we did not compare DKI measurements between different kinds of fitting software. Sixth, we did not evaluate the effect of tumor size or location on DKI measurements. Seventh, we did not conduct histogram analysis even though the technique can be useful in characterizing tumors.

In conclusion, the results of this preliminary study on the utility of non-Gaussian DKI in the assessment of uterine tumors suggest that interobserver agreements in evaluating D, K, and ADC are moderate to excellent; D performs equivalent to conventional DWI in differentiating between benign and malignant uterine tumors; and the mean K of malignant uterine lesions is significantly higher than that of non-tumorous myometrium or endometrium.

## Supporting information

S1 File Study data set. (XLS)

## References

[1] Collins Y, Holcomb K, Chapman-Davis E, Khabele D, Farley JH. Gynecologic cancer disparities: a report from the Health Disparities Taskforce of the Society of Gynecologic Oncology. Gynecol Oncol. 2014;133(2):353–61. doi: 10.1016/j.ygyno.2013.12.039 ; PubMed Central PMCID: PMCPMC4079541. 24406291 10.1016/j.ygyno.2013.12.039 PMC4079541

[2] Siegel RL, Miller KD, Jemal A. Cancer statistics, 2016. CA Cancer J Clin. 2016;66(1):7–30. doi: 10.3322/caac.21332 . 26742998 10.3322/caac.21332

[3] Chen J, Zhang Y, Liang B, Yang Z. The utility of diffusion-weighted MR imaging in cervical cancer. Eur J Radiol. 2010;74(3):e101–6. doi: 10.1016/j.ejrad.2009.04.025 . 19442466 10.1016/j.ejrad.2009.04.025

[4] Hori M, Kim T, Onishi H, Imaoka I, Kagawa Y, Murakami T, et al Endometrial cancer: preoperative staging using three-dimensional T2-weighted turbo spin-echo and diffusion-weighted MR imaging at 3.0 T: a prospective comparative study. Eur Radiol. 2013;23(8):2296–305. doi: 10.1007/s00330-013-2815-0 . 23508278 10.1007/s00330-013-2815-0

[5] Kido A, Fujimoto K, Okada T, Togashi K. Advanced MRI in malignant neoplasms of the uterus. J Magn Reson Imaging. 2013;37(2):249–64. doi: 10.1002/jmri.23716 . 23355429 10.1002/jmri.23716

[6] Kilickesmez O, Bayramoglu S, Inci E, Cimilli T, Kayhan A. Quantitative diffusion-weighted magnetic resonance imaging of normal and diseased uterine zones. Acta Radiol. 2009;50(3):340–7. doi: 10.1080/02841850902735858 . 19235579 10.1080/02841850902735858

[7] Koh DM, Collins DJ. Diffusion-weighted MRI in the body: applications and challenges in oncology. AJR Am J Roentgenol. 2007;188(6):1622–35. doi: 10.2214/AJR.06.1403 . 17515386 10.2214/AJR.06.1403

[8] Kuang F, Ren J, Zhong Q, Liyuan F, Huan Y, Chen Z. The value of apparent diffusion coefficient in the assessment of cervical cancer. Eur Radiol. 2013;23(4):1050–8. doi: 10.1007/s00330-012-2681-1 . 23179520 10.1007/s00330-012-2681-1

[9] Tamai K, Koyama T, Saga T, Morisawa N, Fujimoto K, Mikami Y, et al The utility of diffusion-weighted MR imaging for differentiating uterine sarcomas from benign leiomyomas. Eur Radiol. 2008;18(4):723–30. doi: 10.1007/s00330-007-0787-7 . 17929022 10.1007/s00330-007-0787-7

[10] Bharwani N, Miquel ME, Sahdev A, Narayanan P, Malietzis G, Reznek RH, et al Diffusion-weighted imaging in the assessment of tumour grade in endometrial cancer. Br J Radiol. 2011;84(1007):997–1004. doi: 10.1259/bjr/14980811 ; PubMed Central PMCID: PMCPMC3473695. 21896664 10.1259/bjr/14980811 PMC3473695

[11] Jensen JH, Helpern JA, Ramani A, Lu H, Kaczynski K. Diffusional kurtosis imaging: the quantification of non-gaussian water diffusion by means of magnetic resonance imaging. Magn Reson Med. 2005;53(6):1432–40. doi: 10.1002/mrm.20508 . 15906300 10.1002/mrm.20508

[12] Lu H, Jensen JH, Ramani A, Helpern JA. Three-dimensional characterization of non-gaussian water diffusion in humans using diffusion kurtosis imaging. NMR Biomed. 2006;19(2):236–47. doi: 10.1002/nbm.1020 . 16521095 10.1002/nbm.1020

[13] De Santis S, Gabrielli A, Palombo M, Maraviglia B, Capuani S. Non-Gaussian diffusion imaging: a brief practical review. Magn Reson Imaging. 2011;29(10):1410–6. doi: 10.1016/j.mri.2011.04.006 . 21601404 10.1016/j.mri.2011.04.006

[14] Fieremans E, Jensen JH, Helpern JA. White matter characterization with diffusional kurtosis imaging. Neuroimage. 2011;58(1):177–88. doi: 10.1016/j.neuroimage.2011.06.006 ; PubMed Central PMCID: PMCPMC3136876. 21699989 10.1016/j.neuroimage.2011.06.006 PMC3136876

[15] Kamiya K, Kamagata K, Miyajima M, Nakajima M, Hori M, Tsuruta K, et al Diffusional Kurtosis Imaging in Idiopathic Normal Pressure Hydrocephalus: Correlation with Severity of Cognitive Impairment. Magn Reson Med Sci. 2016;15(3):316–23. doi: 10.2463/mrms.mp.2015-0093 . 26841854 10.2463/mrms.mp.2015-0093 PMC5608128

[16] Pentang G, Lanzman RS, Heusch P, Muller-Lutz A, Blondin D, Antoch G, et al Diffusion kurtosis imaging of the human kidney: a feasibility study. Magn Reson Imaging. 2014;32(5):413–20. doi: 10.1016/j.mri.2014.01.006 . 24582288 10.1016/j.mri.2014.01.006

[17] Quentin M, Pentang G, Schimmoller L, Kott O, Muller-Lutz A, Blondin D, et al Feasibility of diffusional kurtosis tensor imaging in prostate MRI for the assessment of prostate cancer: preliminary results. Magn Reson Imaging. 2014;32(7):880–5. doi: 10.1016/j.mri.2014.04.005 . 24848289 10.1016/j.mri.2014.04.005

[18] Kimura MC, Doring TM, Rueda FC, Tukamoto G, Gasparetto EL. In vivo assessment of white matter damage in neuromyelitis optica: a diffusion tensor and diffusion kurtosis MR imaging study. J Neurol Sci. 2014;345(1–2):172–5. doi: 10.1016/j.jns.2014.07.035 . 25091453 10.1016/j.jns.2014.07.035

[19] Suo S, Chen X, Wu L, Zhang X, Yao Q, Fan Y, et al Non-Gaussian water diffusion kurtosis imaging of prostate cancer. Magn Reson Imaging. 2014;32(5):421–7. doi: 10.1016/j.mri.2014.01.015 . 24602826 10.1016/j.mri.2014.01.015

[20] Tamura C, Shinmoto H, Soga S, Okamura T, Sato H, Okuaki T, et al Diffusion kurtosis imaging study of prostate cancer: preliminary findings. J Magn Reson Imaging. 2014;40(3):723–9. doi: 10.1002/jmri.24379 . 24924835 10.1002/jmri.24379

[21] Umesh Rudrapatna S, Wieloch T, Beirup K, Ruscher K, Mol W, Yanev P, et al Can diffusion kurtosis imaging improve the sensitivity and specificity of detecting microstructural alterations in brain tissue chronically after experimental stroke? Comparisons with diffusion tensor imaging and histology. Neuroimage. 2014;97:363–73. doi: 10.1016/j.neuroimage.2014.04.013 . 24742916 10.1016/j.neuroimage.2014.04.013

[22] Wang JJ, Lin WY, Lu CS, Weng YH, Ng SH, Wang CH, et al Parkinson disease: diagnostic utility of diffusion kurtosis imaging. Radiology. 2011;261(1):210–7. doi: 10.1148/radiol.11102277 . 21771952 10.1148/radiol.11102277

[23] Wu D, Li G, Zhang J, Chang S, Hu J, Dai Y. Characterization of breast tumors using diffusion kurtosis imaging (DKI). PLoS One. 2014;9(11):e113240 doi: 10.1371/journal.pone.0113240 ; PubMed Central PMCID: PMCPMC4236178. 25406010 10.1371/journal.pone.0113240 PMC4236178

[24] Kamagata K, Tomiyama H, Hatano T, Motoi Y, Abe O, Shimoji K, et al A preliminary diffusional kurtosis imaging study of Parkinson disease: comparison with conventional diffusion tensor imaging. Neuroradiology. 2014;56(3):251–8. doi: 10.1007/s00234-014-1327-1 . 24468858 10.1007/s00234-014-1327-1

[25] Kamagata K, Tomiyama H, Motoi Y, Kano M, Abe O, Ito K, et al Diffusional kurtosis imaging of cingulate fibers in Parkinson disease: comparison with conventional diffusion tensor imaging. Magn Reson Imaging. 2013;31(9):1501–6. doi: 10.1016/j.mri.2013.06.009 . 23895870 10.1016/j.mri.2013.06.009

[26] Lawrence EM, Warren AY, Priest AN, Barrett T, Goldman DA, Gill AB, et al Evaluating Prostate Cancer Using Fractional Tissue Composition of Radical Prostatectomy Specimens and Pre-Operative Diffusional Kurtosis Magnetic Resonance Imaging. PLoS One. 2016;11(7):e0159652 doi: 10.1371/journal.pone.0159652 ; PubMed Central PMCID: PMCPMC4965080. 27467064 10.1371/journal.pone.0159652 PMC4965080

[27] Winfield JM, Orton MR, Collins DJ, Ind TE, Attygalle A, Hazell S, et al Separation of type and grade in cervical tumours using non-mono-exponential models of diffusion-weighted MRI. Eur Radiol. 2017;27(2):627–36. doi: 10.1007/s00330-016-4417-0 ; PubMed Central PMCID: PMCPMC5209433. 27221560 10.1007/s00330-016-4417-0 PMC5209433

[28] Nogueira L, Brandao S, Matos E, Nunes RG, Loureiro J, Ramos I, et al Application of the diffusion kurtosis model for the study of breast lesions. Eur Radiol. 2014;24(6):1197–203. doi: 10.1007/s00330-014-3146-5 . 24658871 10.1007/s00330-014-3146-5

[29] Sun K, Chen X, Chai W, Fei X, Fu C, Yan X, et al Breast Cancer: Diffusion Kurtosis MR Imaging-Diagnostic Accuracy and Correlation with Clinical-Pathologic Factors. Radiology. 2015;277(1):46–55. doi: 10.1148/radiol.15141625 . 25938679 10.1148/radiol.15141625

[30] Rosenkrantz AB, Sigmund EE, Johnson G, Babb JS, Mussi TC, Melamed J, et al Prostate cancer: feasibility and preliminary experience of a diffusional kurtosis model for detection and assessment of aggressiveness of peripheral zone cancer. Radiology. 2012;264(1):126–35. doi: 10.1148/radiol.12112290 . 22550312 10.1148/radiol.12112290

[31] Yokosawa S, Sasaki M, Bito Y, Ito K, Yamashita F, Goodwin J, et al Optimization of Scan Parameters to Reduce Acquisition Time for Diffusion Kurtosis Imaging at 1.5T. Magn Reson Med Sci. 2016;15(1):41–8. doi: 10.2463/mrms.2014-0139 . 26104078 10.2463/mrms.2014-0139

